# The *ESR1* gene is associated with risk for canine mammary tumours

**DOI:** 10.1186/1746-6148-9-69

**Published:** 2013-04-10

**Authors:** Kaja Sverdrup Borge, Malin Melin, Patricio Rivera, Stein Istre Thoresen, Matthew Thomas Webster, Henrik von Euler, Kerstin Lindblad-Toh, Frode Lingaas

**Affiliations:** 1 Section of Genetics, Department of Basic Sciences and Aquatic Medicine, Norwegian School of Veterinary Science, P.O Box 8146 Dep., 0033 Oslo, Norway; 2 Science for Life Laboratory, Department of Medical Biochemistry and Microbiology, Uppsala University, Uppsala, Sweden; 3 During the present work: Department of Animal Breeding and Genetics, Uppsala University, Uppsala, Sweden; 4 Currently: Bagarmossen Animal Hospital, Stockholm, Sweden; 5 Section for Clinical Pathology, Department of Basic Sciences and Aquatic Medicine, Norwegian School of Veterinary Science, Oslo, Norway; 6 Department of Clinical Sciences, Swedish University of Agricultural Sciences, Uppsala, Sweden; 7 Broad Institute of Massachusetts Institute of Technology and Harvard, Cambridge, MA, 02139, USA

**Keywords:** Dog, Single nucleotide polymorphism, Allele frequency, Risk, Association, Mammary tumour, Estrogen receptor

## Abstract

**Background:**

The limited within-breed genetic heterogeneity and an enrichment of disease-predisposing alleles have made the dog a very suitable model for the identification of genes associated with risk for specific diseases. Canine mammary cancer is an example of such a disease. However, the underlying inherited risk factors for canine mammary tumours (CMTs) are still largely unknown. In this study, 52 single nucleotide polymorphisms (SNPs) in ten human cancer-associated genes were genotyped in two different datasets in order to identify genes/alleles associated with the development of CMTs. The first dataset consisted of English Springer Spaniel (ESS) CMT cases and controls. ESS is a dog breed known to be at increased risk of developing CMTs. In the second dataset, dogs from breeds known to have a high frequency of CMTs were compared to dogs from breeds with a lower occurrence of these tumours.

**Results:**

We found significant associations to CMT for SNPs and haplotypes in the estrogen receptor 1 (*ESR1*) gene in the ESS material (best *P*_*Bonf*_ = 0.021). A large number of SNPs, among them several SNPs in *ESR1*, showed significantly different allele frequencies between the high and low risk breed groups (best *P*_*Bonf*_ = 8.8E-32, best *P*_*BPerm*_ = 0.076).

**Conclusions:**

The identification of CMT-associated SNPs in *ESR1* in two independent datasets suggests that this gene might be involved in CMT development. These findings also support that CMT may serve as a good model for human breast cancer research.

## Background

The modern dog breeds are a result of vigorous line-breeding and often originate from a few founding ancestors. This has led to extreme phenotypic variation between breeds, but limited genetic variation within breeds [1]. Some breeds have a considerably higher susceptibility to certain diseases than others. This indicates an enrichment of risk alleles within these specific breeds. Canine cancer is an example of such a disease. Mammary tumours are among the most common canine cancer forms [2]. Elderly, intact bitches are primarily affected by these tumours [3,4], with a higher incidence in breeds such as the English Springer Spaniel, Boxer, Cocker Spaniel and Dachshund [3-8]. The differences in breed predisposition clearly indicate a genetic influence on canine mammary tumour (CMT) development. There are many similarities between CMTs and human breast cancer. Among the shared disease characteristics are a spontaneous occurrence of tumours, by which females are primarily affected, and a hormonal influence on tumour development (e.g. oestrogen and progesterone) (as reviewed by Queiroga et al. 2011 [9]). Regional lymph node metastasis seems to be less important as a prognostic factor in dogs than in humans, but metastatic spread is otherwise broadly equal [10]. There are also similarities regarding the histological features and classifications of human breast cancer and CMTs. But while epithelial tumours are by far the most common in humans, CMTs relatively frequently also contain myoepithelial and mesenchymal components. Furthermore studies indicate that CMT and human breast cancer have several mutual prognostic markers and genetic risk factors for disease (as reviewed by Queiroga et al. 2011 [9]). Still, the underlying inherited risk factors for CMTs are largely unknown. Previous studies of genotypes, gene and protein expression in CMTs have identified possible candidate genes and pathways, but the results need to be confirmed or are somewhat inconsistent. However, a study on *BRCA1* and *BRCA2*, two genes well-known to be involved in human breast cancer, showed associations of these genes with mammary tumours in English Springer Spaniels [11]. Another CMT study found mutations in the cancer-associated gene *TP53*[12]. We have previously identified a considerable number of single nucleotide polymorphisms (SNPs) in ten cancer-associated genes known from studies in dogs and/or humans [13]. Some of these SNPs are likely to be associated with canine cancer, and the protein-changing SNPs are of particular interest due to their potential as functional disease-causing variants. In the present study, we aimed at exploring such CMT associations to identify genes involved in the development of mammary tumours in a case-control population of English Springer Spaniels (ESS) and in a second dataset of high and low risk breeds of CMT.

## Results

After quality control, 165 ESS cases and 94 controls were left for analysis (Table 1). Of the 41 SNPs that passed quality control in the ESS case-control dataset, nominal single SNP association was found for two SNPs in *ESR1* exon 2 (*rs21960513*) and intron 7–8 (*ss244244344*) (*P*_*Raw*_ = 0.033 and 0.002, respectively, Table 2). Another SNP in *ESR1* (*ss244244343*) showed borderline nominal significance (*P*_*Raw*_ = 0.052). The risk alleles for the two SNPs *ss244244344* and *ss244244343* are extremely common in the ESS cohort (Table 2). The *ss244244344* and *rs21960513* SNPs were still significant after correcting for multiple testing using 10,000 permutations (*P*_*Perm*_ = 0.018 and 0.042, Table 2). *Ss244244344* also remained significant after applying Bonferroni correction. Of the classified cases, 32 malignant and 78 benign were left after quality control (Table 1). In the comparison of cases with malignant diagnosis vs. controls, *ss244244344* showed nominal significance (*P*_*Raw*_ = 0.016. Additional file 1: Table S3), but the result was not significant after multiple testing correction. We found no significant differences in allele frequencies between benign cases and controls or malignant and benign cases. All association results are provided in Additional file 1.

**Table 1:** Datasets and samples

**Dataset/Breed**	**No. samples**		**CMT grouping**
	**Before QC**^***a***^	**After QC**	
**English Springer Spaniel**^*b*^			
	176	165	Cases
	*83/33*	*78/32*	*Benign/malignant cases*^*c*^
	122	94	Controls
**Canine Biobank dataset**			
Boxer	50	46	High risk
Cocker Spaniel	50	46	High risk
Dachshund	50	48	High risk
English Setter	50	50	High risk
Standard Poodle	50	47	High risk
Beagle	50	47	Low risk
Bernese Mountain Dog	50	49	Low risk
Collie	50	45	Low risk
Shetland Sheepdog	50	50	Low risk

^*a*^ QC: Quality control.

^*b*^ Subset from the study by Rivera et al. [11].

^*c*^ As identified by histopathology by a veterinary pathologist at the time of the genotyping analysis. See Methods for further description.

**Table 2:** **CMT-associated *ESR1 *SNPs in the ESS cases and controls**

**SNP**	**NCBI SNP ID**	**Minor allele**	**Frequency of minor allele**		**Major allele**	***P***_***Raw***_^***a***^	***P***_***Bonf***_^***b***^	***P***_***Perm***_^***c***^	**OR**
			**Cases**	**Controls**					
*ESR1 INT 7-8a*	*ss244244344*	*A*	0.033	0.106	*G*	0.002	0.021	0.018	0.3
*ESR1 EX 2*	*rs21960513*	*C*	0.299	0.391	*T*	0.033	0.362	0.042	0.7
*ESR1 EX 4*	*ss244244343*	*G*	0.006	0.027	*A*	0.052	0.568	0.155	0.2

(n = 165 and n = 94, respectively, after QC).

^*a*^*P*-value from chi-square test in *PLINK*.

^*b*^ Bonferroni corrected for the number of LD blocks (11).

^*c*^ EMP1 value after 10,000 permutations in *PLINK*.

We identified 11 LD blocks with D’ ~ 1 and LOD ≥ 2 in the ESS case-control dataset. One LD block was identified in each of the genes *BRCA2, BRIP1, CDH1, CHEK2, EGFR* and *PTEN*, whereas two and three blocks were found within *ESR1* and *ERBB2 (HER2)*, respectively*.* Only one SNP was genotyped in *BRCA1* and *STK11*, making LD and haplotype analysis impossible. Nominal association was found for one and two haplotypes in each of two *ESR1* LD blocks, respectively, and one haplotype showed borderline nominal significance (Table 3). No haplotypes remained significant after correction for multiple testing. As the total cancer risk of an individual is probably a result of risk alleles at multiple loci, we evaluated the combined effect of the risk haplotypes of the two *ESR1* LD blocks compared to the protective haplotypes. A borderline Bonferroni-significant association was found for the combined risk haplotypes with an odds ratio of 3.3 (*P*_*Bonf*_ = 0.055) (Table 4).

**Table 3:** **CMT-associated *ESR1 *haplotypes in the ESS cases and controls**

**Haplotype**	**Frequency**			***P***_***Raw***_^***a***^	***P***_***Bonf***_^***b***^	***P***_***Perm***_^***c***^	**OR**
	**Total**	**Cases**	**Controls**				
*ESR1 EX 2 and ESR1 EX 4 (rs21960513 and ss244244343)*							
*TA*	0.668	0.701	0.610	0.035	0.381	0.549	1.5
*CA*	0.318	0.293	0.363	0.098	1.000	0.941	0.7
*CG*	0.014	0.006	0.027	0.052	0.572	0.741	0.2
*ESR1 INT 7-8a, –b and ESR1 EX 8 (ss244244344, ss244244345 and ss244244346)*							
*GTG*	0.791	0.818	0.744	0.047	0.517	0.683	1.5
*ACA*	0.091	0.070	0.129	0.024	0.264	0.480	0.5

^*a*^*P*-value from chi-square test in Haploview.

^*b*^ Corrected for the number of LD blocks available (11).

^*c*^ Permutation *P*-value from Haploview, 10,000 permutations.

**Table 4:** **Combined effect of the haplotypes of the two *ESR1 *LD blocks in ESS cases and controls**

**Haplotype combination**	**Frequency**		**Haplotype comparison**	**OR (95% CI)**	***P***_***Raw***_^***a***^	***P***_***Bonf***_^***b***^
	**Cases**	**Controls**				
*TA-GTG* (risk)	207	97	Risk *TA-GTG* vs. rest	1.8 (1.2 - 2.8)	0.007	0.077
			Risk *TA-GTG* vs. protective *CA-ACA*	3.3 (1.4 - 7.9)	0.005	0.055
*CA-ACA* (protective)	9	14	Protective *CA-ACA* vs. rest	0.3 (0.1 - 0.8)	0.010	0.110
Other ^*c*^	56	41				

^*a*^ Fisher-Exact test applied due to low counts of the CG haplotype (less than five in both cases and controls).

^*b*^ Corrected for the number of LD blocks (11).

^*c*^ For 29 cases and 18 controls, the haplotype combinations could not be fully determined due to genotyping failure of one or more of the *ESR1* SNPs.

In the second dataset, 237 dogs of high risk breeds, 191 dogs of low risk breeds and 43 SNPs passed quality control and were included in the study of allele frequencies (Table 1). Nineteen of the 31 SNPs with nominal single SNP associations were significant after 10^7^ permutations, 23 after Bonferroni correction, and among them were the three *ESR1* SNPs *rs21960513*, *ss244244343* and *ss244244344* (Table 5).

**Table 5:** SNPs significantly associated with CMT high and low risk breed groups after Bonferroni correction

**SNP**	**NCBI SNP ID**	**Minor allele**	**Frequency minor allele**		**Major allele**	**OR**	***P***_***Raw***_^***a***^	***P***_***Bonf***_^***b***^	***P***_***Perm***_^***c***^	***P***_***BPerm***_^***d***^
			**High risk breeds**	**Low risk breeds**						
*CHEK2 INT 8-9*	*ss244244336*	*G*	0.269	0.040	*T*	8.8	8.6E-19	7.8E-18	<1.0E-07	0.076
*ESR1 EX 2*	*rs21960513*	*T*	0.629	0.305	*C*	3.9	5.0E-21	4.5E-20	<1.0E-07	0.107
*ERBB2 EX 14*	*rs24537331*	*A*	0.253	0.511	*G*	0.3	8.4E-15	7.6E-14	<1.0E-07	0.110
*BRCA2 INT 18-19*	*ss244244323*	*G*	0.000	0.133	*A*	^_ *e*^	1.8E-16	1.6E-15	<1.0E-07	0.110
*ERBB2 INT 3-4*	*ss244244348*	*G*	0.330	0.681	*A*	0.2	2.4E-24	2.2E-23	<1.0E-07	0.112
*BRCA2 EX 11*	*rs23244160*	*G*	0.174	0.346	*T*	0.4	8.4E-09	7.6E-08	4.2E-05	0.151
*CHEK2 INT 5-6*	*ss244244335*	*G*	0.051	0.000	*A*	^_ *e*^	9.6E-06	8.6E-05	0.005	0.182
*ESR1 EX 4*	*ss244244343*	*G*	0.061	0.394	*A*	0.1	9.8E-33	8.8E-32	<1.0E-07	0.204
*PTEN EX 9*	*ss244244369*	*T*	0.112	0.346	*C*	0.3	1.3E-16	1.2E-15	<1.0E-07	0.209
*BRCA2 EX 5*	*rs23250374*	*C*	0.429	0.23	*T*	2.5	1.1E-09	1.0E-08	1.0E-05	0.321
*ERBB2 INT 12-13b*	*rs24537327*	*A*	0.485	0.283	*G*	2.4	2.2E-09	1.9E-08	2.0E-05	0.362
*ERBB2 EX 13b*	*rs24537329*	*T*	0.486	0.285	*C*	2.4	2.4E-09	2.2E-08	2.1E-05	0.368
*PTEN INT 7-8*	*ss244244368*	*A*	0.183	0.356	*G*	0.5	8.0E-09	7.2E-08	4.0E-05	0.385
*BRCA2 5UTR*	*ss244244322*	*T*	0.343	0.506	*C*	0.5	2.7E-06	2.4E-05	0.002	0.407
*PTEN INT 3-4*	*ss244244367*	*G*	0.191	0.346	*A*	0.6	2.8E-07	2.5E-06	5.0E-04	0.416
*STK11 INT 1-2*	*rs22928814*	*T*	0.241	0.362	*C*	0.6	1.1E-04	1.0E-03	0.031	0.476
*BRIP1 EX 19b*	*ss244244329*	*T*	0.538	0.386	*T*	1.9	9.9E-06	8.9E-05	0.006	0.521
*ERBB2 INT 8-9*	*ss244244349*	*G*	0.089	0.027	*A*	3.2	0.001	5.8E-03	0.101	0.566
*ESR1 INT 7-8a*	*ss244244344*	*A*	0.124	0.223	*G*	0.5	2.3E-04	2.2E-03	0.051	0.708
*BRIP1 INT 8-9a*	*ss244244325*	*G*	0.135	0.011	*A*	14.5	3.2E-11	2.9E-10	<1.0E-07	0.817
*ERBB2 INT 1-2*	*ss244244347*	*T*	0.021	0.000	*C*	^_ *e*^	0.005	0.045	0.340	0.845
*BRIP1 INT 15-16*	*ss244244327*	*T*	0.131	0.011	*A*	14.0	8.0E-11	7.2E-10	<1.0E-07	0.874
*ERBB2 EX13a*	*rs24616607*	*G*	0.044	0.005	*C*	8.5	0.001	5.2E-03	0.095	0.983

(n = 237 and n = 191, respectively, after QC).

^*a*^*P*-value from chi-square test.

^*b*^ Corrected for the number of across-breed LD blocks (9).

^*c*^ EMP1 value after 10^7^ permutations.

^*d*^ EMP1 value after 10,000 breed permutations.

^*e*^ OR could not be calculated as the minor allele was absent in either cases or controls.

However, there were considerable inter-breed variations in SNP allele frequencies for the high and low risk dataset also within the high and low risk breed groups (Table 6). Thus, the mean allele frequency of the group was often not representative for all the breeds included. The overall differences in allele frequency among breeds also caused general inflation of association *P*-values, complicating the interpretation. Breed permutation testing was therefore performed to correct for the inflation. None of the SNPs were significant after breed permutation testing (Table 5).

**Table 6:** Breed-wise average allele frequencies in the high and low risk breed dataset

**SNP**	**Allele frequency (minor allele)**									
	**Minor allele**	**Boxer**	**Cocker Spaniel**	**Dachshund**	**English Setter**	**Standard Poodle**	**Beagle**	**Bernese Mountain Dog**	**Collie**	**Shetland Sheepdog**
*BRCA2 5UTR*	*T*	0.138	0.589	0.682	0.177	0.167	0.244	0.904	0.524	0.330
*BRCA2 EX 5*	*C*	0.130	0.848	0.292	0.160	0.720	0.141	0.684	0.055	0.011
*BRCA2 EX 11*	*G*	0.202	0.141	0.417	0.020	0.100	0.160	0.245	0.556	0.436
*BRCA2 INT 18-19*	*G*	0.000	0.000	0.000	0.000	0.000	0.000	0.000	0.463	0.117
*BRIP1 INT 8-9a*	*G*	0.660	0.000	0.021	0.000	0.010	0.021	0.020	0.000	0.000
*BRIP1 INT 15-16*	*T*	0.652	0.000	0.021	0.000	0.010	0.021	0.020	0.000	0.000
*BRIP1 EX 19b*	*T*	0.872	0.705	0.187	0.120	0.837	0.489	0.204	0.033	0.809
*CHEK2 INT 5-6*	*G*	0.033	0.159	0.073	0.000	0.000	0.000	0.000	0.000	0.000
*CHEK2 INT 8-9*	*G*	0.670	0.120	0.271	0.270	0.050	0.064	0.073	0.000	0.021
*ESR1 EX 2*	*T*	0.967	0.772	0.729	0.410	0.310	0.670	0.102	0.213	0.234
*ESR1 EX 4*	*G*	0.011	0.043	0.000	0.000	0.240	0.053	0.000	0.791	0.766
*ESR1 INT 7-8a*	*A*	0.000	0.083	0.044	0.600	0.000	0.000	0.942	0.000	0.000
*ERBB2 INT1-2*	*T*	0.106	0.000	0.000	0.000	0.000	0.000	0.000	0.000	0.000
*ERBB2 INT 3-4*	*G*	0.033	0.326	0.365	0.720	0.173	0.223	0.918	0.637	0.936
*ERBB2 INT 8-9*	*G*	0.043	0.318	0.052	0.020	0.000	0.109	0.000	0.000	0.000
*ERBB2 INT 12-13b*	*A*	0.915	0.533	0.542	0.240	0.230	0.798	0.071	0.225	0.043
*ERBB2 EX 13a*	*G*	0.223	0.000	0.000	0.000	0.000	0.000	0.020	0.000	0.000
*ERBB2 EX 13b*	*T*	0.926	0.533	0.531	0.240	0.230	0.798	0.071	0.233	0.043
*ERBB2 EX 14*	*A*	0.021	0.250	0.260	0.460	0.260	0.170	0.561	0.451	0.870
*PTEN INT 3-4*	*G*	0.021	0.413	0.031	0.450	0.040	0.128	0.000	0.538	0.734
*PTEN INT 7-8*	*A*	0.000	0.076	0.000	0.470	0.340	0.128	0.010	0.544	0.766
*PTEN EX 9*	*T*	0.000	0.076	0.000	0.450	0.020	0.128	0.000	0.538	0.734
*STK11 INT 1-2*	*T*	0.394	0.348	0.156	0.010	0.310	0.213	0.745	0.467	0.011

To estimate the degree of association seen for the Bonferroni significant polymorphisms, correlation between disease risk and average breed allele frequency for each SNP was calculated. No statistically significant correlations were found.

We applied the LD block criteria D’ ~ 1 and LOD ≥ 2 to the second dataset to study if any of the blocks found in the ESS cases and controls could be re-identified in the high and low risk breeds. Not all blocks were present in all breeds, but a 9kilobase block of eight SNPs in *ERBB2* (*ss244244354, ss244244355, ss244244357, ss244244358, ss244244360, ss244244361, ss244244363* and *ss244244364*) was re-identified for all breeds but the Beagle.

**Figure 1:**
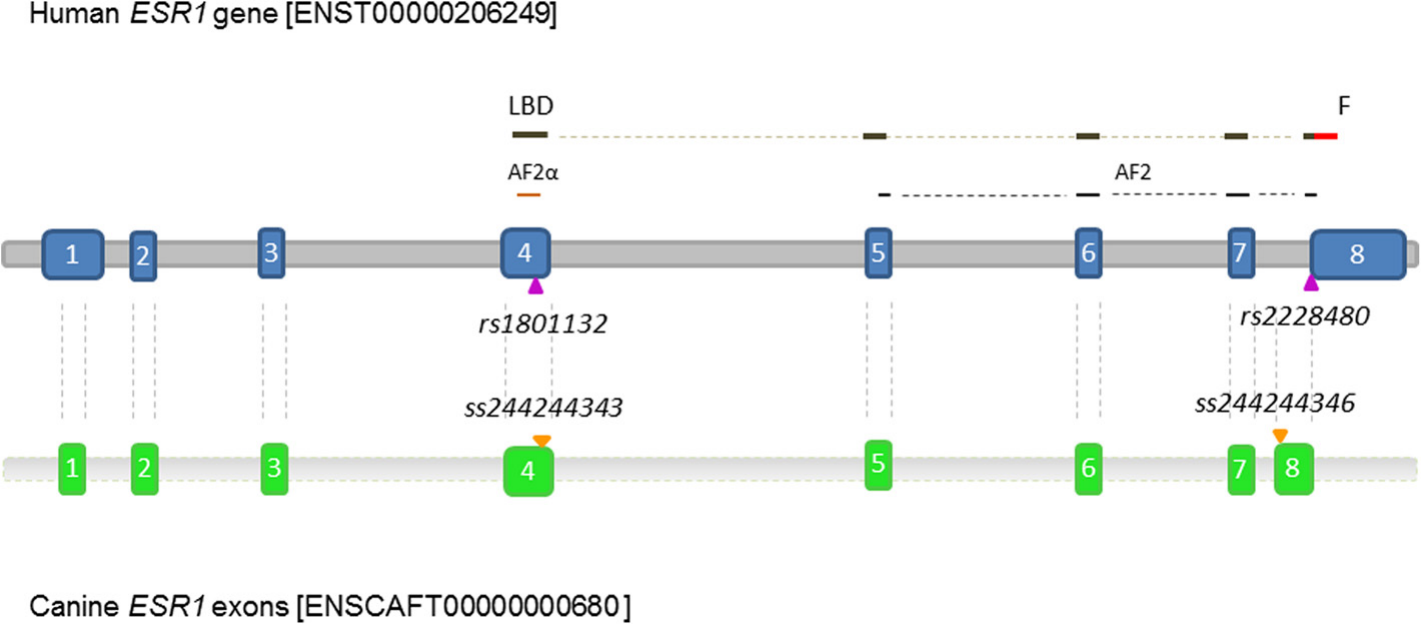
**Schematic illustration of *ESR1 *alignments relative to the human *ESR1 *gene sequence.** The human *ESR1* gene sequence (grey) and exons (blue) with aligned canine *ESR1* exons (green) and human and canine polymorphisms (purple and orange triangles, respectively). The exon numbers are in white letters. Bars above the human exon sequences are indicating the location of the ligand binding domain (LBD, dark brown), F domain (F, red bar), activation factor 2α domain (AF2α, light brown bar) and activation factor 2 domain (AF2, black bars). GATA interacts with the AF2 domain [15-19].

When aligned in Sequencher^®^ the canine *ESR1* exons showed a high match percentage (≥84%) to the human exons, except for exon 1 and 8. We found no human cancer-associated polymorphisms in close proximity to the canine *ESR1* exon 2 SNP (*rs21960513*). However, the canine SNP in exon 4 (*ss244244343*) was positioned one base pair (bp) next to the human *rs1801132* SNP (Figure 1). Another human *ESR1* polymorphism, *rs2228480*, aligned to the canine exon 8 at a position 207 bp downstream from the canine exon 8 SNP (*ss244244346*) (minimum match percentage of 84%) (Figure 1). The canine SNPs *rs21960513* and *ss244244346* were synonymous, while *ss244244343* lead to an amino acid substitution from isoleucine to leucine. Also, *ss244244343* is located in a gene region conserved across four species [13]. However, the substitution was predicted benign and tolerant by PolyPhen and SIFT, respectively [13,14]*.*

## Discussion

Publications about the existence, frequency and importance of CMT-associated germline mutations and their role in the tumour development are sparse. In the present survey, we studied SNPs in known cancer-associated genes and observed significant differences in allele and haplotype frequencies for the *ESR1* gene in the ESS material. These findings were supported by the high and low risk breed groups and suggested an association of *ESR1* alleles with increased risk of CMTs. The *ESR1* gene encodes an estrogen receptor which works as a ligand-activated transcription factor in the cell. Besides its normal role in e.g. sexual development and reproductive function, the estrogen receptor is involved in several pathological processes such as breast cancer (as reviewed by Dahlman-Wright et al. 2006 [20]). Previous studies on human breast cancer have suggested that *ESR1* polymorphisms are associated with the development of these tumours (Additional file 2). The non-synonymous canine exon 4 SNP (*ss244244343*) is positioned one bp downstream compared to the position of the human *rs1801132* SNP. While *rs1801132* is in human codon 325, the canine SNP aligned to a position in human codon 326. Both codons encode amino acids in the hormone binding domain of the human estrogen receptor (Figure 1). This domain is related to receptor dimerization, chaperone binding and recruitment of co-regulators [21]. Studies on *rs1801132* have shown an association with breast malignancies [22,23]. Its *C* allele has been associated with cancer, suggesting that it interferes with the binding of the GATA-1 and GATA-2 transcription factors to the estrogen receptor [21]. GATA transcription factors interact with the activation factor 2 (AF2) region of the ligand binding domain of the human estrogen receptor [15]. There are structural differences in the human and canine ERα proteins, but the major pocket sites seem to be very similar [24]. An association with breast cancer has also been suggested for the human *rs2228480*[25], which aligned in proximity of the synonymous canine exon 8 SNP (*ss244244346*). *Rs2228480* is positioned near the *F* domain of the human estrogen receptor (Figure 1). This domain is believed to be important for the ability of estrogen receptor to distinguish between receptor agonist and antagonist binding [26]. Thus, the observed associations of the canine exon 4 and exon 8 SNPs in our two independent canine datasets are supported by similar effects of closely linked SNPs in the human gene. This suggests *ESR1* as an interesting candidate gene for mammary tumours in dogs as well. Given the prior evidence that *ESR1* plays a role in human breast cancer etiology, it seems probable that the described loci of the present study might be correlated with a causal variant affecting *ESR1* function.

Correction for multiple testing is necessary to adjust for the multiple comparisons performed when testing several SNPs or haplotypes for association. However, SNPs within a gene are very closely linked and not independent observations. Correcting for the number of SNPs would therefore be too conservative. Moreover, this study comprises a selection of biologically important and previously cancer-associated genes where it is likely to find an association, rather than a random set of genes. To assure an appropriate correction for multiple testing of the single SNP and haplotype *P*-values, we therefore used the number of LD blocks. The fact that only one SNP in *ESR1* was significant in the ESS material after Bonferroni correction was somewhat surprising. Possibly, the rest of the SNPs we assessed are not directly causative or in high LD with such variants, creating false negative gene association results. Further, a substantial number of sub-classifications of CMTs exists [27], potentially with different germline mutations contributing to the development of different tumour subtypes and grades of malignancy. Such heterogeneity might complicate the detection of truly cancer-associated mutations. Thus, our study of the ESS dataset might have insufficient power to prove SNP associations to CMT. Still, it has been indicated that about 100 cases and 100 controls should suffice to find loci with strong effects (fivefold) even for complex traits such as cancer [28]. As the one Bonferroni significant *ESR1* SNP (*ss244244344*) is intronic, and we have tested only a limited set of SNPs, this SNP is more likely in LD with a functional mutation than being causative itself. It might also be a false positive. Thus, more studies on the *ESR1* gene are required to establish its role in CMT development.

The considerable breed variations in allele frequencies for the high and low risk dataset might be expected due to between-breed genetic heterogeneity for risk alleles at different risk loci. Yet, the differences are interesting as it appears to be a significant breed-specific accumulation of certain coding variants also in genes that are vital for normal cell function. However, the large allele frequency differences between breeds within the same risk group complicated the interpretation of the results. There is potentially a genetic heterogeneity between breeds as to predominant CMT types and associated risk genes/alleles. If the candidate genes in this study are associated with cancer risk in the selected breeds, the variation in allele frequencies indicates that the associated genes/alleles differ between breeds. Another possibility is that cancer-driving mutations are in the regulatory parts of the genes, and the coding SNPs in the present study can be considered markers for functionally active regulatory sites. During breed differentiation there may have been recombinations between regulatory sites and coding parts resulting in different coding SNPs being linked to regulatory variation in different breeds. Consequently, even if a gene has an important role in cancer development, the associated coding SNP allele/haplotype might vary between breeds. Another challenge is the documentation of high and low risk breeds. As a result of e.g. ancestral patterns of geographical establishment, fluctuations in a dog breed’s popularity and extensive use of popular sires within a country, the genetic composition of a dog breed can change over time and between different geographical locations. Thus, breed predisposition of CMT might vary from subpopulation to subpopulation. We based the selection of high and low risk breeds in this study on previous publications, but not all of them were Norwegian, and some were up to twenty years old. This could possibly be a source of sample error in our study. Moreover, the dogs from the high and low risk breeds in our study are randomly selected without knowledge about the CMT phenotype. They would be a mixture of individuals with high and low risk corresponding to the population frequency of CMT. The frequency and effect of CMT-associated risk alleles would need to be relatively strong to be detected in such a material. It might be that our study is underpowered in that respect. However, we have documented large breed variations in allele frequencies of the coding SNPs in important cancer genes, and there are probably similar differences in the frequency of functionally active haplotypes between dog populations.

## Conclusions

Cancer is a very complex disease. As in human breast malignancies, it is likely that the development of CMTs is influenced by several genes. The identified association of *ESR1* to CMT in the present survey supports the power of the canine model for human breast cancer and the fact that combined studies within and between breeds can add power to the detection of risk alleles also for complex traits. However, the increased risk of CMTs in ESS and other high risk breeds might be due to other SNPs and/or genes than those selected in the present study. There is also a chance that predisposing CMT variants are undetected in our study due to limited power. Nevertheless, this is to our knowledge the first reported association of *ESR1* polymorphisms to CMT and supports *ESR1* as a candidate gene for canine cancer that should be further studied.

## Methods

### Samples

Two separate datasets were included in this study. The first consisted of blood DNA from English Springer Spaniel CMT cases and controls [11] (Table 1). These were privately owned female dogs registered in the Swedish Kennel Club. Approximately half of the dogs were confirmed unrelated at the parental level, while the rest could be as closely related as siblings. A subset of the cases had been classified as malignant (n = 33) or benign (n = 83) CMTs by histopathology by a veterinary pathologist at the time of the genotyping analysis. The rest of the cases were selected based on a veterinary clinical examination confirming the presence of single or multiple nodules within the mammary glands, but they had not (yet) had their mammary tumours surgically removed or histopathologically evaluated. The control dogs were older than eight years with confirmed absence of CMT after palpation of the mammary glands by a veterinarian. However, some of the samples from the material by Rivera et al. were not available for the present study. The same ESS cohort has been genotyped in a parallel study using Illumina 170 K canine HD SNP array, and multidimensional scaling plots were used to evaluate population stratification (data not shown). In this analysis, an outlier group of 29 dogs was identified. These dogs were consequently removed from further analysis in the present study.

The second dataset consisted of EDTA blood samples from the Canine Biobank at the Norwegian School of Veterinary Science (NSVS). In total, this dataset comprised samples from 450 individuals of nine dog breeds (Table 1). The selected breeds were known to be at either high or low risk of developing CMTs according to previous studies. However, the CMT status of the individual dogs from the Canine Biobank was unknown. But according to the higher genetic risk for some of the breeds, an increased allele frequency of associated risk alleles would be expected. Representing breeds at high risk, the Boxer, Cocker Spaniel, Dachshund, English Setter and Standard Poodle were selected. Assumed low risk breeds included in the study were the Beagle, Bernese Mountain Dog, Collie and Shetland Sheepdog [3-8]. Genomic DNA was extracted from the EDTA blood samples using E.Z.N.A Blood Kit according to the manufacturer’s protocol (Omega ^®^, VWR International, West Chester, Pennsylvania, USA). The DNA was analysed for quality and quantity using NanoDrop (Thermo Fisher Scientific, Wilmington, Pennsylvania, USA).

### Single nucleotide polymorphism selection and genotyping

All samples were genotyped for 52 previously described canine SNPs [13] (Additional file 3). These SNPs were located in ten genes previously reported to be cancer-associated in humans; *BRCA1*, *BRCA2*, *BRIP1*, *CDH1*, *CHEK2*, *EGFR*, *ERBB2 (HER2)*, *ESR1*, *PTEN* and *STK11* (Table 7). Twenty of the SNPs were found in coding regions, including 11 synonymous and 9 non-synonymous SNPs. The SNPs were distributed into two pools and genotyped using the Sequenom iPLEX Gold Mass ARRAY^®^ according to the manufacturer’s protocol (Sequenom^®^, San Diego, California, USA). The genotyping was performed at Broad Institute, Cambridge, Massachusetts.

**Table 7:** Genes evaluated for association to CMT

**Gene**	**Human chromosome**	**Canine chromosome**	**No. SNPs genotyped**	**Location of SNPs (Mb) (CanFam 2.0)**
*BRCA1*	17	9	1	23.306
*BRCA2*	13	25	5	10.782-10.732
*BRIP1*	17	9	5	38.191-38.304
*CDH1*	16	5	5	83.792-83.771
*CHEK2*	22	26	3	25.104-25.086
*EGFR*	7	18	5	8.982-9.027
*ERBB2 (HER2)*	17	9	18	26.106-26.088
*ESR1*	6	1	5	45.129-45.409
*PTEN*	10	26	4	40.921-40.978
*STK11*	19	20	1	60.709

### Single SNP and haplotype association analysis

Single SNP and haplotype analysis were performed separately for the two different study datasets; the ESS cases were compared to the controls, and the high risk breeds from the Canine Biobank were all compared to the low risk breeds. Single SNP association analysis was also performed for the subset of ESS cases with benign tumours vs. controls, malignant tumours vs. controls and benign vs. malignant tumours. Only samples and SNPs with a genotyping success rate of ≥75% and SNPs with a minor allele frequency (MAF) ≥1% were included in the single SNP and haplotype association analysis. The *PLINK* software [29,30] was used for analysing allele frequencies, single *χ*^2^ SNP association and SNP odds ratios. Haploview [31,32] was used to identify LD blocks with a D’ ~ 1 and LOD ≥ 2 for each dataset and to generate haplotypes and haplotype association statistics. Odds ratios for haplotypes at each specific locus were estimated using calculators at VassarStats [33]. The nominal (raw) *χ*^2^*P*-values from the single SNP and haplotype analysis were Bonferroni corrected using the number of LD blocks to adjust for the problem of multiple comparisons that arises from evaluating several SNPs or haplotypes. Multiple testing correction using 10,000 permutations for the ESS dataset and 10^7^ permutations for the high and low risk breed dataset was also performed. Further, we did permutation testing by permuting the high/low risk labels simultaneously for all dogs in each breed in combination with PLINK analysis of association, using 10,000 permutations. A *P*-value of less than 0.05 after correction for multiple testing was reckoned statistically significant.

Considering each individual breed as the study unit rather than the individual dog, we performed pairwise correlation analysis for the high and low risk breed dataset using JMP 8 (SAS Institute Inc., North Carolina, USA). The correlation between the disease risk (high or low) and average allele frequency for each breed for each SNP was estimated, regarding a *P*-value of less than 0.05 statistically significant. Bonferroni correction was performed using the number of LD blocks across breeds (9).

Known human breast cancer-associated *ESR1* polymorphisms (Additional file 2) were compared to the canine SNPs in this study by alignment using the Sequencher software (Gene Codes Corporation, Ann Arbor, MI, USA). The full genomic human *ESR1* gene sequence [ENST00000206249] was aligned to the canine [ENSCAFT00000000680, (CanFam2.0)] [16] and human exon sequences and polymorphisms with flanking sequence.

## Abbreviations

BRCA1: Breast cancer 1, early onset; BRCA2: Breast cancer 2, early onset; BRIP1: BRCA1 interacting protein C-terminal helicase 1; CDH1: Cadherin 1, type 1, E-cadherin (epithelial); CHEK2: Checkpoint kinase 2; CMT: Canine mammary tumour; D’: Normalized measure of linkage disequilibrium; EGFR: Epidermal growth factor receptor; ESR1: Estrogen receptor 1; ERBB2 (HER2): v-erb-b2 erythroblastic leukemia viral oncogene homolog 2, neuro/glioblastoma derived oncogene homolog (avian); ESS: English Springer Spaniel; MAF: Minor allele frequency; LD: Linkage disequilibrium; NSVS: Norwegian School of Veterinary Science; PTEN: Phosphatase and tensin homolog; SNP: Single nucleotide polymorphism; nsSNP: Non-synonymous SNP; STK11: Serine/threonine kinase 11.

## Competing interests

The authors declare that they have no competing interests.

## Authors’ contributions

KSB helped conceive the study and its design, carried out the DNA isolation, SNP and haplotype association analysis, sequence alignments and drafted the manuscript. MM contributed to the SNP and haplotype association analysis and revised the manuscript. PR and HVE collected the ESS case and control samples and planned CMT analysis. SIT was responsible for the collection of samples for the Canine Biobank and revised the manuscript. MTW helped with statistical analysis and performed the breed permutations. KLT contributed to the SNP and haplotype association analysis and revised the manuscript. FL conceived the study and its design, contributed to the SNP and haplotype association analysis and revised the manuscript. All authors read and approved the final manuscript.

## Supplementary Material

Additional file 1 All results from ESS single SNP and haplotype association analysis. Click here for file

Additional file 2 **Cancer-associated human *ESR1 *polymorphisms that were compared to the canine SNPs.** Click here for file

Additional file 3 Genotyped single nucleotide polymorphisms, genome position, reference ID and alleles. Click here for file

## References

[B1] Patterson DF Companion animal medicine in the age of medical genetics J Vet Intern Med 2000 14 1 9 10.1111/j.1939-1676.2000.tb01492.x 10668810

[B2] Bronden LB Nielsen SS Toft N Kristensen AT Data from the Danish veterinary cancer registry on the occurrence and distribution of neoplasms in dogs in Denmark Vet Rec 2010 166 586 590 10.1136/vr.b4808 20453236

[B3] Egenvall A Bonnett BN Ohagen P Olson P Hedhammar A von Euler H Incidence of and survival after mammary tumors in a population of over 80,000 insured female dogs in Sweden from 1995 to 2002 Prev Vet Med 2005 69 109 127 10.1016/j.prevetmed.2005.01.014 15899300

[B4] Boldizsar H Szenci O Muray T Csenki J Studies on canine mammary tumours. I. Age, seasonal and breed distribution Acta Vet Hung 1992 40 75 87 1476093

[B5] Arnesen K Gamlem H Glattre E Grøndalem J Moe L Nordstoga K The Norwegian canine cancer register 1990–1998. Report from the project "Cancer in the dog" EJCAP 2001 11 159 169

[B6] Dahl K Moe L Indrebø I Gamlem H Forekomst av mammatumor hos beslektede boxere [Occurence of mammary tumor in related Boxers] Nor Vet Tidsskr 2002 114 615 622

[B7] Priester WA Occurrence of mammary neoplasms in bitches in relation to breed, age, tumour type, and geographical region from which reported J Small Anim Pract 1979 20 1 11 10.1111/j.1748-5827.1979.tb07014.x 759718

[B8] Moe L Population-based incidence of mammary tumours in some dog breeds J Reprod Fertil Suppl 2001 57 439 443 11787188

[B9] Queiroga FL Raposo T Carvalho MI Prada J Pires I Canine mammary tumours as a model to study human breast cancer: most recent findings In Vivo 2011 25 455 465 21576423

[B10] Owen LN A comparative study of canine and human breast cancer Invest Cell Pathol 1979 2 257 275 396282

[B11] Rivera P Melin M Biagi T Fall T Haggstrom J Lindblad-Toh K von Euler H Mammary tumor development in dogs is associated with BRCA1 and BRCA2 Cancer Research 2009 69 8770 8774 10.1158/0008-5472.CAN-09-1725 19887619

[B12] Veldhoen N Watterson J Brash M Milner J Identification of tumour-associated and germ line p53 mutations in canine mammary cancer Br J Cancer 1999 81 409 415 10.1038/sj.bjc.6690709 10507764 PMC2362910

[B13] Borge KS Borresen-Dale AL Lingaas F Identification of genetic variation in 11 candidate genes of canine mammary tumour Vet Comp Oncol 2011 9 241 250 10.1111/j.1476-5829.2010.00250.x 22077404

[B14] Ng PC Henikoff S Predicting deleterious amino acid substitutions Genome Res 2001 11 863 874 10.1101/gr.176601 11337480 PMC311071

[B15] Jones DR Schmidt RJ Pickard RT Foxworthy PS Eacho PI Estrogen receptor-mediated repression of human hepatic lipase gene transcription J Lipid Res 2002 43 383 391 11893774

[B16] Ensembl release 67 [http://www.ensembl.org/index.html]

[B17] Norris JD Fan D Kerner SA McDonnell DP Identification of a third autonomous activation domain within the human estrogen receptor Mol Endocrinol 1997 11 747 754 10.1210/me.11.6.747 9171238

[B18] Herynk MH Fuqua SA Estrogen receptor mutations in human disease Endocr Rev 2004 25 869 898 10.1210/er.2003-0010 15583021

[B19] Kumar R Zakharov MN Khan SH Miki R Jang H Toraldo G Singh R Bhasin S Jasuja R The dynamic structure of the estrogen receptor J Amino Acids 2011 2011 812540 22312471 10.4061/2011/812540 PMC3268042

[B20] Dahlman-Wright K Cavailles V Fuqua SA Jordan VC Katzenellenbogen JA Korach KS Maggi A Muramatsu M Parker MG Gustafsson JA International Union of Pharmacology. LXIV. Estrogen receptors Pharmacol Rev 2006 58 773 781 10.1124/pr.58.4.8 17132854

[B21] Anghel A Raica M Narita D Seclaman E Nicola T Ursoniu S Anghel M Popovici E Estrogen receptor alpha polymorphisms: correlation with clinicopathological parameters in breast cancer Neoplasma 2010 57 306 315 20429621 10.4149/neo_2010_04_306

[B22] Vasconcelos A Medeiros R Veiga I Pereira D Carrilho S Palmeira C Azevedo C Lopes CS Analysis of estrogen receptor polymorphism in codon 325 by PCR-SSCP in breast cancer: association with lymph node metastasis Breast J 2002 8 226 229 10.1046/j.1524-4741.2002.08407.x 12100115

[B23] Kallel I Rebai M Khabir A Farid NR Rebai A Genetic polymorphisms in the EGFR (R521K) and estrogen receptor (T594T) genes, EGFR and ErbB-2 protein expression, and breast cancer risk in Tunisia J Biomed Biotechnol 2009 2009 753683 19636371 10.1155/2009/753683 PMC2711625

[B24] Toniti W Suthiyotha N Puchadapirom P Jenwitheesuk E Binding capacity of ER-alpha ligands and SERMs: comparison of the human, dog and cat Asian Pac J Cancer Prev 2011 12 2875 2879 22393957

[B25] Tapper W Hammond V Gerty S Ennis S Simmonds P Collins A Eccles D The influence of genetic variation in 30 selected genes on the clinical characteristics of early onset breast cancer Breast Cancer Res 2008 10 R108 10.1186/bcr2213 19094228 PMC2656905

[B26] Montano MM Muller V Trobaugh A Katzenellenbogen BS The carboxy-terminal F domain of the human estrogen receptor: role in the transcriptional activity of the receptor and the effectiveness of antiestrogens as estrogen antagonists Mol Endocrinol 1995 9 814 825 10.1210/me.9.7.814 7476965

[B27] Goldschmidt M Pena L Rasotto R Zappulli V Classification and grading of canine mammary tumors Vet Pathol 2011 48 117 131 10.1177/0300985810393258 21266722

[B28] Karlsson EK Lindblad-Toh K Leader of the pack: gene mapping in dogs and other model organisms Nat Rev Genet 2008 9 713 725 18714291 10.1038/nrg2382

[B29] Purcell S Neale B Todd-Brown K Thomas L Ferreira MA Bender D Maller J Sklar P de Bakker PI Daly MJ Sham PC PLINK: a tool set for whole-genome association and population-based linkage analyses Am J Hum Genet 2007 81 559 575 10.1086/519795 17701901 PMC1950838

[B30] PLINK (version 1.07) [http://pngu.mgh.harvard.edu/purcell/plink/]

[B31] Barrett JC Fry B Maller J Daly MJ Haploview: analysis and visualization of LD and haplotype maps Bioinformatics 2005 21 263 265 10.1093/bioinformatics/bth457 15297300

[B32] Haploview [http://www.broadinstitute.org/haploview]

[B33] VassarStats:Website for statistical computation [http://vassarstats.net]

