# Supplementary material for: The ESR1 gene is associated with risk for canine mammary tumours
Source: BMC Vet Res. 2013 Apr 10;9:69. doi: 10.1186/1746-6148-9-69 (PMC3637093; doi:10.1186/1746-6148-9-69)
Supplement: Additional file 1 — All results from ESS single SNP and haplotype association analysis. [file 1746-6148-9-69-S1.docx]

**Table S1. Single SNP association analysis results for SNPs that passed QC in the ESS case-control dataset.**

|  |  |  | **Frequency of minor allele** | |  |  |  |  |  |
| --- | --- | --- | --- | --- | --- | --- | --- | --- | --- |
| **SNP** | **NCBI SNP ID** | **Minor allele** | **Cases** | **Controls** | **Major allele** | ***P_raw_^a^*** | ***P_Bonf_^b^*** | ***P_Perm_^c^*** | **OR** |
| *BRCA1 EX 9* | *ss244244321* | *G* | 0.112 | 0.122 | *A* | 0.727 | 7.994 | 0.772 | 0.91 |
| *BRCA2 5UTR* | *ss244244322* | *C* | 0.503 | 0.483 | *T* | 0.667 | 7.341 | 0.682 | 1.08 |
| *BRCA2 EX 5* | *rs23250374* | *C* | 0.412 | 0.42 | *T* | 0.857 | 9.431 | 0.924 | 0.97 |
| *BRCA2 EX 11* | *rs23244160* | *G* | 0.408 | 0.415 | *T* | 0.878 | 9.658 | 0.883 | 0.97 |
| *BRIP1 INT 8-9a* | *ss244244325* | *G* | 0.045 | 0.032 | *A* | 0.453 | 4.978 | 0.486 | 1.44 |
| *BRIP1 INT 8-9b* | *ss244244326* | *C* | 0.055 | 0.027 | *T* | 0.138 | 1.514 | 0.215 | 2.11 |
| *BRIP1 INT 15-16* | *ss244244327* | *T* | 0.046 | 0.032 | *A* | 0.445 | 4.891 | 0.434 | 1.45 |
| *BRIP1 EX 19a* | *ss244244328* | *G* | 0.1 | 0.059 | *A* | 0.103 | 1.137 | 0.142 | 1.79 |
| *BRIP1 EX 19b* | *ss244244329* | *C* | 0.055 | 0.027 | *T* | 0.134 | 1.475 | 0.189 | 2.13 |
| *CDH1 INT 1-2* | *ss244244330* | *C* | 0.097 | 0.085 | *T* | 0.654 | 7.197 | 0.758 | 1.15 |
| *CDH1 EX 5* | *ss244244331* | *T* | 0.013 | 0.017 | *C* | 0.761 | 8.374 | 0.985 | 0.79 |
| *CDH1 INT 5-6* | *ss244244332* | *G* | 0.094 | 0.086 | *C* | 0.764 | 8.405 | 0.795 | 1.10 |
| *CDH1 INT 12-13a* | *ss244244333* | *G* | 0.094 | 0.085 | *A* | 0.737 | 8.102 | 0.752 | 1.12 |
| *CDH1 INT 12-13b* | *ss244244334* | *T* | 0.094 | 0.085 | *C* | 0.737 | 8.102 | 0.752 | 1.12 |
| *CHEK2 INT 5-6* | *ss244244335* | *G* | 0.138 | 0.108 | *A* | 0.566 | 6.222 | 0.576 | 1.32 |
| *CHEK2 INT 8-9* | *ss244244336* | *G* | 0.087 | 0.138 | *T* | 0.316 | 3.479 | 0.339 | 0.60 |
| *EGFR INT 23-24a* | *ss244244340* | *C* | 0.024 | 0.032 | *T* | 0.605 | 6.651 | 1.0 | 0.75 |
| *EGFR INT 24-25* | *ss244244342* | *G* | 0.024 | 0.032 | *A* | 0.605 | 6.651 | 1.0 | 0.75 |
| *ERBB2 INT 3-4* | *ss244244348* | *G* | 0.121 | 0.117 | *A* | 0.888 | 9.764 | 0.893 | 1.04 |
| *ERBB2 INT 8-9* | *ss244244349* | *G* | 0.055 | 0.048 | *A* | 0.743 | 8.168 | 0.852 | 1.15 |
| *ERBB2 INT 12-13a* | *ss244244352* | *A* | 0.312 | 0.277 | *C* | 0.396 | 4.355 | 0.426 | 1.19 |
| *ERBB2 INT 12-13b* | *rs24537327* | *G* | 0.352 | 0.337 | *A* | 0.734 | 8.077 | 0.743 | 1.07 |
| *ERBB2 EX 13b* | *rs24537329* | *C* | 0.364 | 0.346 | *T* | 0.683 | 7.511 | 0.703 | 1.08 |
| *ERBB2 EX 14* | *rs24537331* | *A* | 0.045 | 0.065 | *G* | 0.336 | 3.692 | 0.324 | 0.68 |
| *ERBB2 INT 14-15* | *ss244244354* | *T* | 0.312 | 0.261 | *C* | 0.216 | 2.375 | 0.221 | 1.29 |
| *ERBB2 INT 15-16* | *ss244244355* | *T* | 0.312 | 0.271 | *C* | 0.328 | 3.609 | 0.364 | 1.22 |
| *ERBB2 INT 16-17* | *ss244244357* | *G* | 0.311 | 0.271 | *A* | 0.342 | 3.762 | 0.364 | 1.21 |
| *ERBB2 INT 18-19* | *ss244244358* | *G* | 0.323 | 0.283 | *C* | 0.356 | 3.920 | 0.346 | 1.21 |
| *ERBB2 EX 23* | *ss244244360* | *C* | 0.312 | 0.271 | *T* | 0.328 | 3.609 | 0.364 | 1.22 |
| *ERBB2 INT 23-24* | *ss244244361* | *T* | 0.315 | 0.271 | *C* | 0.294 | 3.238 | 0.312 | 1.24 |
| *ERBB2 EX 27a* | *ss244244363* | *A* | 0.312 | 0.269 | *G* | 0.301 | 3.312 | 0.309 | 1.23 |
| *ERBB2 EX 27b* | *ss244244364* | *T* | 0.312 | 0.271 | *C* | 0.328 | 3.609 | 0.364 | 1.22 |
| *ESR1 EX 2* | *rs21960513* | *C* | 0.299 | 0.391 | *T* | 0.033 | 0.362 | 0.042 | 0.66 |
| *ESR1 EX 4* | *ss244244343* | *G* | 0.006 | 0.027 | *A* | 0.052 | 0.568 | 0.155 | 0.22 |
| *ESR1 INT 7-8a* | *ss244244344* | *A* | 0.033 | 0.106 | *G* | 0.002 | 0.021 | 0.018 | 0.29 |
| *ESR1 INT 7-8b* | *ss244244345* | *C* | 0.156 | 0.207 | *T* | 0.143 | 1.571 | 0.155 | 0.71 |
| *ESR1 EX 8* | *ss244244346* | *A* | 0.181 | 0.239 | *G* | 0.120 | 1.321 | 0.181 | 0.70 |
| *PTEN INT 3-4* | *ss244244367* | *G* | 0.171 | 0.245 | *A* | 0.290 | 3.193 | 0.301 | 0.63 |
| *PTEN INT 7-8* | *ss244244368* | *A* | 0.078 | 0.143 | *G* | 0.208 | 2.287 | 0.218 | 0.51 |
| *PTEN EX 9* | *ss244244369* | *T* | 0.075 | 0.138 | *C* | 0.203 | 2.228 | 0.209 | 0.50 |
| *STK11 INT 1-2* | *rs22928814* | *T* | 0.142 | 0.117 | *C* | 0.413 | 4.546 | 0.437 | 1.25 |

*^a^ P*-value from chi-square test in *PLINK*.

*^b^* Bonferroni corrected for the number of LD blocks (11).

*^c^* EMP1 value after 10.000 permutations in *PLINK*.

**Table S2. Results for haplotype association analysis for the ESS case-control dataset.**

| **Haplotype** | **Frequency** | | | **χ^2^** | ***P_Raw_****^a^* | ***P_Bonf_****^b^* | ***P_Perm_****^c^* | **OR** |
| --- | --- | --- | --- | --- | --- | --- | --- | --- |
|  | **Total** | **Case** | **Controls** |  |  |  |  |  |
| *BRCA2 EX 11 and EX 5 (rs23244160 and rs23250374)* | | | |  |  |  |  |  |
| *TC* | 0.413 | 0.412 | 0.415 | 0.004 | 0.949 | 10.44 | 1.00 | 0.99 |
| *GT* | 0.409 | 0.409 | 0.409 | 0.000 | 0.995 | 10.95 | 1.00 | 1.00 |
| *TT* | 0.176 | 0.179 | 0.171 | 0.058 | 0.810 | 8.91 | 1.00 | 1.06 |
| *BRIP1 EX 19a and –b (ss244244328 and ss244244329)* | | | | |  |  |  |  |
| *AT* | 0.915 | 0.900 | 0.941 | 2.652 | 0.103 | 1.14 | 0.96 | 0.56 |
| *GC* | 0.044 | 0.055 | 0.027 | 2.205 | 0.138 | 1.51 | 0.98 | 2.10 |
| *GT* | 0.041 | 0.045 | 0.032 | 0.564 | 0.453 | 4.98 | 1.00 | 1.43 |
| *CDH1 INT 12-13b, INT 12-13a, INT 5-6, EX 5 and INT 1-2 (ss244244334, ss244244333, ss244244332, ss244244331 and ss244244330)* | | | | | | | | |
| *CACCT* | 0.907 | 0.903 | 0.915 | 0.200 | 0.654 | 7.20 | 1.00 | 0.86 |
| *TGGCC* | 0.069 | 0.072 | 0.064 | 0.107 | 0.744 | 8.18 | 1.00 | 1.13 |
| *TGGTC* | 0.022 | 0.022 | 0.021 | 0.009 | 0.924 | 10.17 | 1.00 | 1.05 |
| *CHEK2 INT 8-9 and INT 5-6 (ss244244336 and ss244244335)* | | | | |  |  |  |  |
| *TA* | 0.544 | 0.544 | 0.543 | 0.000 | 0.992 | 10.91 | 1.00 | 1.00 |
| *GA* | 0.255 | 0.242 | 0.277 | 0.736 | 0.391 | 4.30 | 1.00 | 0.83 |
| *TG* | 0.201 | 0.214 | 0.180 | 0.845 | 0.358 | 3.94 | 1.00 | 1.24 |
| *EGFR2 INT 23-24a and EGFR INT 24-25 (ss244244340 and ss244244342)* | | | | | |  |  |  |
| *TA* | 0.973 | 0.976 | 0.968 | 0.268 | 0.605 | 6.65 | 1.00 | 1.34 |
| *CG* | 0.027 | 0.024 | 0.032 | 0.268 | 0.605 | 6.65 | 1.00 | 0.74 |
| *ERBB2 INT 12-13a, INT 12-13b and INT 8-9 (rs24537327, ss244244352 and ss244244349)* | | | | | | |  |  |
| *ACA* | 0.595 | 0.588 | 0.607 | 0.183 | 0.669 | 7.35 | 1.00 | 0.92 |
| *GAA* | 0.299 | 0.312 | 0.277 | 0.721 | 0.396 | 4.35 | 1.00 | 1.18 |
| *GCA* | 0.054 | 0.046 | 0.069 | 1.241 | 0.265 | 2.92 | 1.00 | 0.65 |
| *ACG* | 0.049 | 0.053 | 0.04 | 0.430 | 0.512 | 5.63 | 1.00 | 1.34 |
| *ERBB2 EX 14 and EX 13b (rs24537331 and rs24537329)* | | | |  |  |  |  |  |
| *GT* | 0.643 | 0.636 | 0.654 | 0.167 | 0.683 | 7.51 | 1.00 | 0.92 |
| *GC* | 0.304 | 0.318 | 0.28 | 0.845 | 0.358 | 3.94 | 1.00 | 1.20 |
| *AC* | 0.053 | 0.045 | 0.066 | 1.027 | 0.311 | 3.42 | 1.00 | 0.67 |
| *ERBB2 SNP INT 14-15, INT 15-16, INT 16-17, INT 18-19, EX 23, INT 23-24, EX 27a, EX 27b (ss244244354, ss244244355, ss244244357, ss244244358, ss244244360, ss244244361, ss244244363 and ss244244364)* | | | | | | | | |
| *CGCTCACC* | 0.699 | 0.685 | 0.723 | 0.844 | 0.358 | 3.94 | 1.00 | 0.83 |
| *TATCGGTT* | 0.293 | 0.312 | 0.261 | 1.531 | 0.216 | 2.37 | 1.00 | 1.28 |
| *ESR1 EX 2 and ESR1 EX 4 (rs21960513 and ss244244343)* | | | | |  |  |  |  |
| *TA* | 0.668 | 0.701 | 0.610 | 4.465 | 0.035 | 0.38 | 0.57 | 1.50 |
| *CA* | 0.318 | 0.293 | 0.363 | 2.734 | 0.098 | 1.08 | 0.94 | 0.73 |
| *CG* | 0.014 | 0.006 | 0.027 | 3.789 | 0.052 | 0.57 | 0.74 | 0.22 |
| *ESR1 INT 7-8a and -b and ESR1 EX 8 (ss244244344, ss244244345 and ss244244346)* | | | | | | |  |  |
| *GTG* | 0.791 | 0.818 | 0.744 | 3.944 | 0.047 | 0.52 | 0.68 | 1.55 |
| *ACA* | 0.091 | 0.070 | 0.129 | 5.131 | 0.024 | 0.26 | 0.48 | 0.51 |
| *GCA* | 0.084 | 0.088 | 0.078 | 0.132 | 0.716 | 7.88 | 1.00 | 1.14 |
| *GTA* | 0.034 | 0.025 | 0.049 | 2.023 | 0.155 | 1.70 | 0.99 | 0.50 |
| *PTEN INT 3-4, INT 7-8 and PTEN EX 9 (ss244244367, ss244244368 and ss244244369)* | | | | | | |  |  |
| *AGC* | 0.648 | 0.648 | 0.647 | 0.002 | 0.965 | 10.61 | 1.00 | 1.00 |
| *GAT* | 0.263 | 0.255 | 0.277 | 0.301 | 0.583 | 6.42 | 1.00 | 0.89 |
| *GGC* | 0.076 | 0.079 | 0.071 | 0.094 | 0.759 | 8.35 | 1.00 | 1.12 |
| *AAC* | 0.012 | 0.015 | 0.006 | 0.970 | 0.325 | 3.57 | 1.00 | 2.52 |

*^a^ P*-value from chi-square test in Haploview.

*^b^* Corrected for the number of LD blocks available (11).

*^c^* Permutation *P*-value from Haploview, 10.000 permutations.

**Table S3. Single SNP association analysis results for different subsets in the ESS case-control dataset***^a^***.**

|  |  | **Frequency** | | | **Malignant vs benign** | | **Malignant vs controls** | | **Benign vs controls** | |
| --- | --- | --- | --- | --- | --- | --- | --- | --- | --- | --- |
| **SNP** | **Minor allele** | **Malignant** | **Benign** | **Controls** | ***P_Raw_****^b^* | ***EMP1****^c^* | ***P_Raw_****^b^* | ***EMP1****^c^* | ***P_Raw_****^b^* | ***EMP1****^c^* |
| *BRCA1 EX 9* | *G* | 0.203 | 0.096 | 0.122 | 0.086 | 0.144 | 0.111 | 0.120 | 0.441 | 0.492 |
| *BRCA2 5UTR* | *C* | 0.500 | 0.486 | 0.483 | 0.629 | 0.601 | 0.815 | 0.802 | 0.952 | 0.970 |
| *BRCA2 EX 5* | *C* | 0.406 | 0.397 | 0.420 | 0.671 | 0.761 | 0.845 | 0.886 | 0.669 | 0.748 |
| *BRCA2 EX 11* | *G* | 0.403 | 0.435 | 0.415 | 0.464 | 0.485 | 0.871 | 0.888 | 0.707 | 0.745 |
| *BRIP1 INT 8-9a* | *G* | 0.031 | 0.064 | 0.032 | 0.330 | 0.504 | 0.979 | 1.000 | 0.158 | 0.186 |
| *BRIP1 INT 8-9b* | *C* | 0.047 | 0.051 | 0.027 | 0.892 | 1.000 | 0.424 | 0.677 | 0.232 | 0.292 |
| *BRIP1 INT 15-16* | *T* | 0.031 | 0.065 | 0.032 | 0.321 | 0.341 | 0.979 | 1.000 | 0.150 | 0.148 |
| *BRIP1 EX 19a* | *G* | 0.078 | 0.115 | 0.059 | 0.412 | 0.479 | 0.578 | 0.764 | 0.059 | 0.074 |
| *BRIP1 EX 19b* | *C* | 0.048 | 0.051 | 0.027 | 0.930 | 1.000 | 0.398 | 0.449 | 0.232 | 0.292 |
| *CDH1 INT 1-2* | *C* | 0.094 | 0.090 | 0.085 | 0.925 | 1.000 | 0.832 | 1.000 | 0.879 | 1.000 |
| *CDH1 EX 5* | *T* | 0.036 | - | 0.017 | - | - | 0.387 | 0.481 | - | - |
| *CDH1 INT 5-6* | *G* | 0.094 | 0.083 | 0.086 | 0.803 | 1.000 | 0.851 | 1.000 | 0.929 | 0.927 |
| *CDH1 INT 12-13a* | *G* | 0.094 | 0.083 | 0.085 | 0.803 | 1.000 | 0.832 | 1.000 | 0.953 | 1.000 |
| *CDH1 INT 12-13b* | *T* | 0.094 | 0.083 | 0.085 | 0.803 | 1.000 | 0.832 | 1.000 | 0.953 | 1.000 |
| *CHEK2 INT 5-6* | *G* | 0.095 | 0.122 | 0.108 | 0.743 | 0.778 | 0.871 | 0.921 | 0.806 | 0.812 |
| *CHEK2 INT 8-9* | *G* | 0.125 | 0.064 | 0.138 | 0.434 | 0.381 | 0.893 | 0.912 | 0.218 | 0.239 |
| *EGFR INT 23-24a* | *C* | 0.031 | 0.026 | 0.032 | 0.817 | 1.000 | 0.979 | 1.000 | 0.730 | 1.000 |
| *EGFR INT 24-25* | *G* | 0.031 | 0.026 | 0.032 | 0.817 | 1.000 | 0.979 | 1.000 | 0.730 | 1.000 |
| *ERBB2 INT 3-4* | *G* | 0.172 | 0.109 | 0.117 | 0.204 | 0.268 | 0.261 | 0.321 | 0.815 | 0.874 |
| *ERBB2 INT 8-9* | *G* | 0.047 | 0.038 | 0.048 | 0.775 | 1.000 | 0.974 | 1.000 | 0.671 | 0.803 |
| *ERBB2 INT 12-13a* | *A* | 0.375 | 0.282 | 0.277 | 0.301 | 0.347 | 0.139 | 0.160 | 0.911 | 1.000 |
| *ERBB2 INT 12-13b* | *G* | 0.403 | 0.327 | 0.337 | 0.455 | 0.492 | 0.348 | 0.377 | 0.842 | 0.844 |
| *ERBB2 EX 13b* | *C* | 0.406 | 0.346 | 0.346 | 0.594 | 0.655 | 0.384 | 0.461 | 0.994 | 1.000 |
| *ERBB2 EX 14* | *A* | 0.031 | 0.051 | 0.065 | 0.517 | 0.718 | 0.311 | 0.323 | 0.586 | 0.542 |
| *ERBB2 INT 14-15* | *T* | 0.375 | 0.282 | 0.261 | 0.301 | 0.347 | 0.082 | 0.113 | 0.656 | 0.714 |
| *ERBB2 INT 15-16* | *T* | 0.375 | 0.282 | 0.271 | 0.301 | 0.347 | 0.117 | 0.153 | 0.824 | 0.900 |
| *ERBB2 INT 16-17* | *G* | 0.371 | 0.282 | 0.271 | 0.283 | 0.324 | 0.136 | 0.153 | 0.824 | 0.900 |
| *ERBB2 INT 18-19* | *G* | 0.387 | 0.296 | 0.283 | 0.332 | 0.341 | 0.128 | 0.141 | 0.799 | 0.806 |
| *ERBB2 EX 23* | *C* | 0.375 | 0.282 | 0.271 | 0.301 | 0.347 | 0.117 | 0.153 | 0.824 | 0.900 |
| *ERBB2 INT 23-24* | *T* | 0.375 | 0.289 | 0.271 | 0.349 | 0.434 | 0.117 | 0.153 | 0.724 | 0.801 |
| *ERBB2 EX 27a* | *A* | 0.375 | 0.282 | 0.269 | 0.301 | 0.347 | 0.109 | 0.113 | 0.785 | 0.806 |
| *ERBB2 EX 27b* | *T* | 0.375 | 0.282 | 0.271 | 0.301 | 0.347 | 0.117 | 0.153 | 0.824 | 0.900 |
| *ESR1 EX 2* | *C* | 0.281 | 0.318 | 0.391 | 0.590 | 0.665 | 0.115 | 0.124 | 0.163 | 0.201 |
| *ESR1 EX 4* | *G* | 0.016 | 0.006 | 0.027 | - | - | 0.619 | 0.762 | 0.155 | 0.308 |
| *ESR1 INT 7-8a* | *A* | 0.000 | 0.048 | 0.106 | 0.116 | 0.452 | ***0.016*** | 0.084 | 0.070 | 0.194 |
| *ESR1 INT 7-8b* | *C* | 0.156 | 0.171 | 0.207 | 0.790 | 0.847 | 0.371 | 0.492 | 0.396 | 0.431 |
| *ESR1 EX 8* | *A* | 0.156 | 0.204 | 0.239 | 0.414 | 0.507 | 0.169 | 0.275 | 0.446 | 0.551 |
| *PTEN INT 3-4* | *G* | 0.333 | 0.195 | 0.245 | 0.278 | 0.299 | 0.495 | 0.505 | 0.563 | 0.582 |
| *PTEN INT 7-8* | *A* | 0.177 | 0.091 | 0.143 | 0.347 | 0.357 | 0.734 | 0.742 | 0.428 | 0.459 |
| *PTEN EX 9* | *T* | 0.177 | 0.089 | 0.138 | 0.331 | 0.352 | 0.693 | 0.700 | 0.442 | 0.478 |
| *STK11 INT 1-2* | *T* | 0.094 | 0.141 | 0.117 | 0.610 | 0.666 | 0.609 | 0.663 | 0.507 | 0.532 |

*^a^*None of the SNPs were significant after Bonferroni correction (results not shown).

*^b^P*-value from chi-square test in *PLINK*.

*^c^*EMP1 value after 10.000 permutations in *PLINK*.
