# Supplementary material for: The ESR1 gene is associated with risk for canine mammary tumours
Source: BMC Vet Res. 2013 Apr 10;9:69. doi: 10.1186/1746-6148-9-69 (PMC3637093; doi:10.1186/1746-6148-9-69)
Supplement: Additional file 2 — Cancer-associated human ESR1 polymorphisms that were compared to the canine SNPs. [file 1746-6148-9-69-S2.pdf]

Supplementary file 2. Cancer-associated human ESR1 polymorphisms that were compared to the canine SNPs in the present study.

SNP ID	Position (bp)[a]	Alleles	Exon/intron[b]	Reference
Rs3734805	151939350	*A/C/G/T*	Intron 1-2	[1]
ESR1 1002	152126824	*T/G*	Intron 1-2	[2]
Rs2077647	152129077	*A/G*	Intron 1-2	[2]
Rs3778609	152133187	*C/T*	Intron 1-2	[3]
Rs12665044	152149872	*C/T*	Intron 1-2	[3]
Rs827421	152157122	*C /T*	Intron 1-2	[2] [3]
Rs2234693	152163335	*C/T*	Intron 1-2	[4]
Rs9340799	152163381	*A/G*	Intron 1-2	[5]
Rs1801132	152265522	*C/G*	Intron 3-4	[2] [6] [7]
Rs3020314	152270672	*C/T*	Intron 3-4	[8]
Rs7739506	152286443	*A/G*	Intron 4	[3]
Rs7766585	152396036	*G/T*	Intron 6-7	[9]
Rs2228480	152420095	*A/G*	Intron 7-8	[8] [10] [11]
Rs3798577	152421130	*C/T*	Exon 8	[10]
Rs2747648	152422335	*C/T*	Exon 8	[12]
Rs1062577	152423905	*A/T*	Exon 8	[8]
Rs9383938	151987357	*G/T*	Intron 1-2	[1]

[a] Position in GRCh37[13]
[b] Exon/intron position according to transcript ENST00000206249[14].

References

1. Fletcher O, Johnson N, Orr N, Hosking FJ, Gibson LJ, Walker K, Zelenika D, Gut I, Heath S, Palles C, Coupland B, Broderick P, Schoemaker M, Jones M, Williamson J, Chilcott-Burns S, Tomczyk K, Simpson G, Jacobs KB, Chanock SJ, Hunter DJ, Tomlinson IP, Swerdlow A, Ashworth A, Ross G, dos SS, I, Lathrop M, Houlston RS, Peto J: **Novel breast cancer susceptibility locus at 9q31.2: results of a genome-wide association study.** *J Natl Cancer Inst* 2011, 103:425-435.

2. Gold B, Kalush F, Bergeron J, Scott K, Mitra N, Wilson K, Ellis N, Huang H, Chen M, Lippert R, Halldorsson BV, Woodworth B, White T, Clark AG, Parl FF, Broder S, Dean M, Offit K: **Estrogen receptor genotypes and haplotypes associated with breast cancer risk.** *Cancer Res* 2004, 64:8891-8900.

3. Ding SL, Yu JC, Chen ST, Hsu GC, Hsu HM, Ho JY, Lin YH, Chang CC, Fann CS, Cheng CW, Wu PE, Shen CY: **Diverse associations between ESR1 polymorphism and breast cancer development and progression.** *Clin Cancer Res* 2010, 16:3473-3484.

4. Zhang B, Beeghly-Fadiel A, Lu W, Cai Q, Xiang YB, Zheng Y, Long J, Ye C, Gu K, Shu XO, Gao Y, Zheng W: **Evaluation of functional genetic variants for breast cancer risk: results from the Shanghai breast cancer study.** *Am J Epidemiol* 2011, 173:1159-1170.

5. Wang J, Higuchi R, Modugno F, Li J, Umblas N, Lee J, Lui LY, Ziv E, Tice JA, Cummings SR, Rhees B: **Estrogen receptor alpha haplotypes and breast cancer risk in older Caucasian women.** *Breast Cancer Res Treat* 2007, 106:273-280.

6. Wedren S, Lovmar L, Humphreys K, Magnusson C, Melhus H, Syvanen AC, Kindmark A, Landegren U, Fermer ML, Stiger F, Persson I, Baron J, Weiderpass E: **Oestrogen receptor alpha gene haplotype and postmenopausal breast cancer risk: a case control study.** *Breast Cancer Res* 2004, 6:R437-R449.

7. Siddig A, Mohamed AO, Awad S, Hassan AH, Zilahi E, Al-Haj M, Bernsen R, Adem A: **Estrogen receptor alpha gene polymorphism and breast cancer.** *Ann N Y Acad Sci* 2008, 1138:95-107.

8. Li N, Dong J, Hu Z, Shen H, Dai M: **Potentially functional polymorphisms in ESR1 and breast cancer risk: a meta-analysis.** *Breast Cancer Res Treat* 2010, 121:177-184.

9. Harlid S, Ivarsson MI, Butt S, Hussain S, Grzybowska E, Eyfjord JE, Lenner P, Forsti A, Hemminki K, Manjer J, Dillner J, Carlson J: **A candidate CpG SNP approach identifies a breast cancer associated ESR1-SNP.** *Int J Cancer* 2011, 129:1689-1698.

10. Anghel A, Raica M, Narita D, Seclaman E, Nicola T, Ursoniu S, Anghel M, Popovici E: **Estrogen receptor alpha polymorphisms: correlation with clinicopathological parameters in breast cancer.** *Neoplasma* 2010, 57:306-315.

11. Kallel I, Rebai M, Khabir A, Farid NR, Rebai A: **Genetic polymorphisms in the EGFR (R521K) and estrogen receptor (T594T) genes, EGFR and ErbB-2 protein expression, and breast cancer risk in Tunisia.** *J Biomed Biotechnol* 2009, 2009:753683.

12. Tchatchou S, Jung A, Hemminki K, Sutter C, Wappenschmidt B, Bugert P, Weber BH, Niederacher D, Arnold N, Varon-Mateeva R, Ditsch N, Meindl A, Schmutzler RK, Bartram CR, Burwinkel B: **A variant affecting a putative miRNA target site in estrogen**

receptor (ESR) 1 is associated with breast cancer risk in premenopausal women. *Carcinogenesis* 2009, 30:59-64.

13. **NCBI Entrez SNP** [http://www.ncbi.nlm.nih.gov/snp/]

14. **Ensembl release 67** [http://www.ensembl.org/index.html]
