# Supplementary material for: The ESR1 gene is associated with risk for canine mammary tumours
Source: BMC Vet Res. 2013 Apr 10;9:69. doi: 10.1186/1746-6148-9-69 (PMC3637093; doi:10.1186/1746-6148-9-69)
Supplement: Additional file 3 — Genotyped single nucleotide polymorphisms, genome position, reference ID and alleles. [file 1746-6148-9-69-S3.docx]

**Supplementary file 1. Genotyped single nucleotide polymorphisms, genome position, reference ID and alleles**.

| **Gene** | **Exon/intron position** | **CFA** | **Position (bp)***^a^* | **SNP ID***^a^* | **SNP type** | **Alleles** |
| --- | --- | --- | --- | --- | --- | --- |
| ***BRCA1*** | EX 9 | 9 | 23.306.247 | *ss244244321* | Synonymous | *G/A* |
| ***BRCA2*** | 5’UTR | 25 | 10.782.592 | *ss244244322* | Non-coding | *C/T* |
|  | EX 5 | 25 | 10.771.796 | *rs23250374* | Non-synonymous | *T/C* |
|  | EX 11 | 25 | 10.759.790 | *rs23244160* | Non-synonymous | *T/G* |
|  | INT 18-19 | 25 | 10.735.321 | *ss244244323* | Non-coding | *A/G* |
|  | EX 24 | 25 | 10.732.072 | *ss244244324* | Non-synonymous | *A/G* |
| ***BRIP1*** | INT 8-9a | 9 | 38.191.393 | *ss244244325* | Non-coding | *A/G* |
|  | INT 8-9b | 9 | 38.191.453 | *ss244244326* | Non-coding | *C/T* |
|  | INT 15-16 | 9 | 38.279.741 | *ss244244327* | Non-coding | *A/T* |
|  | EX 19a | 9 | 38.304.647 | *ss244244328* | Non-synonymous | *G/A* |
|  | EX 19b | 9 | 38.304.788 | *ss244244329* | Non-synonymous | *C/T* |
| ***CDH1*** | INT 1-2 | 5 | 83.792.310 | *ss244244330* | Non-coding | *T/C* |
|  | EX 5 | 5 | 83.784.803 | *ss244244331* | Synonymous | *C/T* |
|  | INT 5-6 | 5 | 83.784.684 | *ss244244332* | Non-coding | *C/G* |
|  | INT 12-13a | 5 | 83.771.216 | *ss244244333* | Non-coding | *A/G* |
|  | INT 12-13b | 5 | 83.771.075 | *ss244244334* | Non-coding | *C/T* |
| ***CHEK2*** | INT 5-6 | 26 | 25.104.233 | *ss244244335* | Non-coding | *A/G* |
|  | INT 8-9 | 26 | 25.094.678 | *ss244244336* | Non-coding | *T/G* |
|  | INT 13-14 | 26 | 25.086.320 | *ss244244337* | Non-coding | *A/G* |
| ***EGFR*** | EX 2a | 18 | 8.982.529 | *rs9206306* | Non-synonymous | *G/A* |
|  | EX 2b | 18 | 8.982.559 | *ss244244338* | Non-synonymous | *C/T* |
|  | INT 23-24a | 18 | 9.027.221 | *ss244244340* | Non-coding | *T/C* |
|  | INT 23-24b | 18 | 9.027.235 | *ss244244341* | Non-coding | *C/T* |
|  | INT 24-25 | 18 | 9.027.308 | *ss244244342* | Non-coding | *A/G* |
| ***ESR1*** | EX 2 | 1 | 45.176.696 | *rs21960513* | Synonymous | *T/C* |
|  | EX 4 | 1 | 45.254.192 | *ss244244343* | Non-synonymous | *A/G* |
|  | INT 7-8a | 1 | 45.405.038 | *ss244244344* | Non-coding | *G/A* |
|  | INT 7-8b | 1 | 45.405.045 | *ss244244345* | Non-coding | *T/C* |
|  | EX 8 | 1 | 45.409.598 | *ss244244346* | Synonymous | *G/A* |
| ***ERBB2 (HER2)*** | INT 1-2 | 9 | 26.106.227 | *ss244244347* | Non-coding | *C/T* |
|  | INT 3-4 | 9 | 26.104.296 | *ss244244348* | Non-coding | *A/G* |
|  | INT 8-9 | 9 | 26.101.298 | *ss244244349* | Non-coding | *A/G* |
|  | EX9 | 9 | 26.101.119 | *ss244244350* | Non-synonymous | *A/G* |
|  | INT 11-12 | 9 | 26.098.955 | [*rs24550703*](http://www.ensembl.org/Canis_familiaris/Variation/Summary?db=core;g=ENSCAFG00000016351;r=9:26088707-26112823;source=dbSNP;t=ENSCAFT00000025936;v=rs24550703;vf=4341477) | Non-coding | *T/C* |
|  | INT 12-13a | 9 | 26.098.462 | *ss244244352* | Non-coding | *C/A* |
|  | INT 12-13b | 9 | 26.098.331 | [*rs24537327*](http://www.ensembl.org/Canis_familiaris/Variation/Summary?db=core;g=ENSCAFG00000016351;r=9:26088707-26112823;source=dbSNP;t=ENSCAFT00000025936;v=rs24537327;vf=4327909) | Non-coding | *A/G* |
|  | EX13a | 9 | 26.098.200 | *rs24616607* | Synonymous | *G/C* |
|  | EX 13b | 9 | 26.098.149 | [*rs24537329*](http://www.ensembl.org/Canis_familiaris/Variation/Summary?db=core;g=ENSCAFG00000016351;r=9:26088707-26112823;source=dbSNP;t=ENSCAFT00000025936;v=rs24537329;vf=4327911) | Synonymous | *T/C* |
|  | EX 14 | 9 | 26.097.964 | *rs24537331* | Synonymous | *G/A* |
|  | INT 14-15 | 9 | 26.097.871 | *ss244244354* | Non-coding | *C/T* |
|  | INT 15-16 | 9 | 26.094.521 | *ss244244355* | Non-coding | *C/T* |
|  | INT 16-17 | 9 | 26.094.315 | *ss244244357* | Non-coding | *A/G* |
|  | INT 18-19 | 9 | 26.092.406 | *ss244244358* | Non-coding | *C/G* |
|  | EX 23 | 9 | 26.090.739 | *ss244244360* | Synonymous | *T/C* |
|  | INT 23-24 | 9 | 26.090.622 | *ss244244361* | Non-coding | *C/T* |
|  | EX 27a | 9 | 26.089.004 | *ss244244363* | Synonymous | *G/A* |
|  | EX 27b | 9 | 26.088.731 | *ss244244364* | Synonymous | *C/T* |
| ***PTEN*** | INT2-3 | 26 | 40.921.779 | *ss244244366* | Non-coding | *A/G* |
|  | INT 3-4 | 26 | 40.921.979 | *ss244244367* | Non-coding | *A/G* |
|  | INT 7-8 | 26 | 40.974.966 | *ss244244368* | Non-coding | *G/A* |
|  | EX 9 | 26 | 40.978.713 | *ss244244369* | Synonymous | *C/T* |
| ***STK 11*** | INT 1-2 | 20 | 60.709.778 | [*rs22928814*](http://www.ensembl.org/Canis_familiaris/Variation/Summary?db=core;g=ENSCAFG00000019538;r=20:60701050-60719499;source=dbSNP;v=rs22928814;vf=2958648) | Non-coding | *C/T* |

*^a^*According to NCBI Entrez SNP[1] (CanFam2.1)

**References**

1. **NCBI Entrez SNP** [<http://www.ncbi.nlm.nih.gov/snp/>]
